# Religion and HIV in Tanzania: influence of religious beliefs on HIV stigma, disclosure, and treatment attitudes

**DOI:** 10.1186/1471-2458-9-75

**Published:** 2009-03-04

**Authors:** James Zou, Yvonne Yamanaka, Muze John, Melissa Watt, Jan Ostermann, Nathan Thielman

**Affiliations:** 1 School of Engineering and Applied Sciences, Harvard University, Cambridge, MA, USA; 2 Pratt School of Engineering, Duke University, Durham, NC, USA; 3 Selian AIDS Control Program, Selian Hospital, Arusha, Tanzania; 4 University of North Carolina at Chapel Hill, School of Public Health, Chapel Hill, NC, USA; 5 Center for Health Policy, Duke University, Durham, NC, USA; 6 Department of Medicine, Division of Infectious Diseases and International Health, Duke University, Durham, NC, USA

## Abstract

**Background:**

Religion shapes everyday beliefs and activities, but few studies have examined its associations with attitudes about HIV. This exploratory study in Tanzania probed associations between religious beliefs and HIV stigma, disclosure, and attitudes toward antiretroviral (ARV) treatment.

**Methods:**

A self-administered survey was distributed to a convenience sample of parishioners (n = 438) attending Catholic, Lutheran, and Pentecostal churches in both urban and rural areas. The survey included questions about religious beliefs, opinions about HIV, and knowledge and attitudes about ARVs. Multivariate logistic regression analysis was performed to assess how religion was associated with perceptions about HIV, HIV treatment, and people living with HIV/AIDS.

**Results:**

Results indicate that shame-related HIV stigma is strongly associated with religious beliefs such as the belief that HIV is a punishment from God (p < 0.01) or that people living with HIV/AIDS (PLWHA) have not followed the Word of God (p < 0.001). Most participants (84.2%) said that they would disclose their HIV status to their pastor or congregation if they became infected. Although the majority of respondents (80.8%) believed that prayer could cure HIV, almost all (93.7%) said that they would begin ARV treatment if they became HIV-infected. The multivariate analysis found that respondents' hypothetical willingness to begin ARV treatme was not significantly associated with the belief that prayer could cure HIV or with other religious factors. Refusal of ARV treatment was instead correlated with lack of secondary schooling and lack of knowledge about ARVs.

**Conclusion:**

The decision to start ARVs hinged primarily on education-level and knowledge about ARVs rather than on religious factors. Research results highlight the influence of religious beliefs on HIV-related stigma and willingness to disclose, and should help to inform HIV-education outreach for religious groups.

## Background

Religious activities, communities, and beliefs frame the daily behaviors and attitudes of many people living in countries with high rates of HIV/AIDS. Tanzania, with an estimated HIV prevalence rate of 6.5% in 2005 [1], is no exception. Christians and Muslims each make up 30% to 40% of the population. The majority of the remaining population are members of indigenous religions [2]. However, despite the public health community's widespread interest in understanding and addressing HIV-related issues such as stigma and antiretroviral (ARV) treatment adherence, relatively little is known about the influence that religion and religious communities have on people's attitudes and practices concerning HIV/AIDS.

Several previous studies have called attention to the correlation between religion and behaviors that help to protect against contracting HIV. Much of the research in this area has focused on Muslim populations in African countries. It has been suggested that several of the religiously motivated behaviors practiced by Muslims are favorable for HIV prevention and have led to lower HIV prevalence rates among Muslims [3]. These factors include higher rates of circumcision, fewer self-reported instances of extramarital sexual intercourse [4], and reduced consumption of alcohol (decreasing high-risk sexual activity) [5]. It has also been argued that the Pentecostal church's emphasis on salvation and strong social presence (e.g. youth groups, frequent prayer meetings) prevents members from engaging in as much extra- and pre- marital sex as other Christian denominations, thus protecting against HIV [6]. However, strong religious beliefs do not always correlate with HIV protective behaviors. In a rural region of Senegal, Muslims and Catholics who considered religion "very important" were less likely to display HIV-preventive attitudes (e.g. intentions to change behavior to protect against contracting HIV) than those who attached less importance to religion [7]. In other studies, religious affiliation has been found to correlate with level of HIV knowledge [8,9], but not necessarily with protective behaviors [8].

On the community level, religious organizations are influential social networks that have the power to support or stigmatize people living with HIV/AIDS (PLWHA), promote or impede HIV education, and endorse or reject medical treatment of HIV. In Tanzania and other countries with high rates of HIV, faith based organizations (FBOs) are major providers of HIV/AIDS care, service, and education [10]. Churches can give people support for both spiritual matters and daily material needs. They can provide PLWHA with spiritual counseling, prayers for healing, hope for personal spiritual salvation, social and material support, personalized care when they are sick, and assurances of burial after they die [11]. On a regulatory level, some churches require or heavily encourage couples to be tested for HIV before getting married [12,13].

The sexual and moral connotations frequently associated with HIV transmission can also turn the church into a stigmatizing atmosphere for PLWHA. The perceived stigma occurs at all levels, from church leaders to congregation members [14]. Many of the stigmatizing attitudes towards PLWHA arise from people's beliefs that PLWHA have behaved immorally and fears of acquiring HIV through casual contact with PLWHA [15]. This latter category of HIV stigma can be further broken down into stigma arising from shame about HIV and stigma arising from the tendency to attach blame to PLWHA [16]. Shame stigma is also important to understand in the context of organized religion. Shame about HIV is closely linked to internalized, self-directed stigmatization [17], which can lead PLWHA to withdraw from social settings such as their religious community. Fears of stigmatization and blame are also closely linked to disclosure intentions [18-20].

Religious beliefs significantly shape individuals' outlooks on living with HIV. Faith practices and beliefs can provide a sense of peace and hope, and can also help people to prepare for and accept death [14]. People often turn to religion to make sense of and come to terms with being HIV-infected. Prayer, meditation, faith in God, and other forms of religious participation have frequently been cited by PLWHA in Tanzania and other African countries as major strategies for coping with HIV/AIDS [21]. Studies conducted in the United States have found that PLWHA use religion to cope with their illness [22,23], that being diagnosed with HIV often strengthens people's faith [22,24], that an increase in spirituality/religiousness after being diagnosed with HIV is correlated with slower disease progression [24], and that spiritual beliefs about HIV influence end-of-life decisions [25].

Religious beliefs about HIV can also contribute to fatalistic attitudes and passive resignation, which hinders participation in treatment. In one study from rural Mali, people who believed that AIDS was a punishment from God had more fatalistic attitudes (e.g. agreeing to the statement "I believe that if a person has HIV/AIDS most treatments will not change anything") than those who did not [26]. The belief that prayer can cure HIV may also challenge adherence to antiretroviral (ARV) treatment programs. A study on ARV adherence in Uganda found that 6 out of 558 (1.2%) patients discontinued their treatment because they believed that their pastors' prayers had cured them of HIV [27].

The studies discussed above provide a sound starting point for understanding the connections between religion and beliefs about HIV. However, almost no previous work has been done to elucidate the specific aspects of religious belief that influence perceptions about HIV and PLWHA. Therefore, the aim of this study was to examine how religious beliefs and church environments influence HIV-related stigma and beliefs about the causes and possible treatments of HIV. Specifically, religious and demographic factors across both urban and rural Tanzanians belonging to the three largest Christian denominations in Tanzania (Catholic, Lutheran, Pentecostal) were compared across key indicators of shame-related HIV stigma, intentions of disclosing serostatus to the religious community if HIV-infected, and willingness to begin ARV treatment if HIV-infected. The questions examined here are relevant to both social and clinical applications, as they provide a better understanding of how religious beliefs and environments relate to stigmatizing behavior towards PLWHA, beliefs about HIV transmission and treatment, and other attitudes about HIV/AIDS. This information can help guide collaborations between church leaders and clinicians or HIV educators, and can be used to more effectively incorporate people's religious beliefs into outreach or treatment plans.

## Methods

### Survey Design and Administration

A 43 question self-administered written survey in Swahili was constructed to probe religious beliefs, stigma, and knowledge related to HIV. The survey was informed by interviews and focus group discussions with key religious informants. The survey was administered during June and July of 2007.

Prior to data collection at any church, the study team met with the church leader to explain the purpose of the research and seek his approval. Congregants were introduced to the study by an announcement made by a Tanzanian research assistant during the church services. The surveys were completed individually and on-site in approximately 20 minutes. Research assistants were available to answer any participant questions. Participants returned their completed surveys to the research assistants.

The administration procedure differed slightly at the Pentecostal church in Arusha. The pastor felt that it was inappropriate for a person who was not a member of the church to perform the survey in his congregation. Instead, the pastor read the introductory script during the service and distributed and collected the surveys.

Surveys were completed anonymously to protect the privacy of the participants. Congregants were told that participation in the survey was completely voluntary and that they could leave questions that they did not feel comfortable answering blank. Informed consent was therefore implied by taking a survey, completing it, and returning it. The Duke University Institutional Review Board approved the protocol for this study.

### Sample

Surveys were distributed to and collected from the congregations of Catholic, Lutheran, and Pentecostal churches in both the large urban town of Arusha and a small rural village in the Singe ward of the Babati region. Six churches were sampled; one from each denomination at each site. Both of the Pentecostal churches were Tanzanian Assemblies of God. In Arusha the largest church of each denomination was selected. In Singe the sole church of each denomination was selected. Survey distribution and collection were performed during a single Sunday worship service at all but one of the churches. At the Pentecostal church in Arusha surveying was done during the Tuesday evening fellowship meeting. The surveys were distributed to a convenience sample of eligible (>18 years old) volunteers. All eligible congregants were given the opportunity to take the survey.

### Independent Variables

The first section of the survey measured basic demographic characteristics including sex, age, level of schooling completed, marital status, frequency of church attendance, and religious denomination. Rural or urban residency was assigned based on survey site.

Several Yes/No and Agree/Disagree/Neutral questions were used to measure religious beliefs relating to HIV. Participants were asked if they believed that HIV is a punishment from God, if they believed that an HIV-infected person has not followed the Word of God, and if they believed that prayer could cure HIV.

Knowledge, attitudes, and practices relating to HIV and ARVs were assessed using a combination of individual questions and binary index variables (hereafter referred to as indices). Indices were constructed to measure respondents' knowledge about ARVs and fear of HIV transmission by casual contact. The individual questions contained in each index are shown in Table 1. Respondents were classified as having ARV knowledge if they replied "Yes" to at least one of the ARV knowledge questions listed in Table 1. Similarly, respondents were classified as "fearing casual contact HIV transmission" if they replied "Yes" to at least one of the questions addressing casual contact HIV transmission fears (Table 1). The questions about casual contact HIV transmission fears were previously field tested in Tanzanian cohorts [16], and were chosen for use in the present study because they address means of contact that commonly occur in church settings (sitting next to fellow congregants, sharing a communion cup, etc.). Participants were also asked if they had ever been tested for HIV and about their frequency of condom use. Participants were not asked about their HIV status.

**Table 1:** Index definitions.

**INDEX**	**QUESTIONS**
*HIV-Related Shame Stigma*	▪ People with HIV should be ashamed of themselves (Agree/Neutral/Disagree)
	▪ I would be ashamed if someone in my family had HIV/AIDS (Agree/Neutral/Disagree)
	▪ I would feel ashamed if I was infected with HIV (Agree/Neutral/Disagree)

*Fear of Casual Contact HIV Transmission*	▪ Would you be afraid of getting HIV if you cared for a person living with HIV? (Yes/No)
	▪ Would you be afraid of getting HIV if you sat next to an HIV+ person? (Yes/No)
	▪ Would you be afraid of getting HIV if you were exposed to the sweat of an HIV+ person? (Yes/No)
	▪ Would you be afraid of getting HIV if you were exposed to the saliva of an HIV+ person? (Yes/No)

*Disclosure to the Religious Community*	▪ If you were HIV+, would you tell your pastor? (Yes/No)
	▪ If you were HIV+ would you want your congregation to know? (Yes/No)

*ARV Knowledge*	▪ Do you know where ARVs are provided? (Yes/No)
	▪ Can an HIV+ person get free ARVs in Arusha/Babati? (Yes/No/Don't Know)
	▪ Do you believe that an HIV+ person can become healthier by taking ARVs? (Yes/No/Don't Know)

Questions composing the indices for shame-related HIV stigma, casual contact HIV transmission fear, disclosure to religious community, and ARV knowledge.

### Outcome Variables

Three aspects of HIV were identified as outcome (dependent) variables for assessing how religion is associated with people's perceptions of HIV, PLWHA, and ARV treatment. They were: (1) shame-related HIV stigma, (2) willingness to disclose HIV status to the religious community if HIV-infected and (3) willingness to begin doctor-recommended ARV treatment if HIV-infected. These indicators were chosen because of their relevance to HIV/AIDS education and outreach in both the religious and medical sectors.

A three question index was constructed to measure shame-related HIV stigma. Respondents were classified as expressing shame-related HIV stigma if they replied "Agree" to at least one of the shame-related HIV stigma statements listed in Table 1. In previous Tanzanian field tests this set of three questions was found to be a reliable (α = 0.8) measure capturing the shame dimension of HIV-related stigma [16].

Similarly, respondents were classified as being willing to disclose to the religious community if they replied "Yes" to at least one of the religious community disclosure questions shown in Table 1.

Willingness to begin ARV treatment was measured by a single Yes/No question, "If you tested positive for HIV, would you start ARV treatment if doctors recommended it?"

### Statistical Analysis

Chi-square analysis was performed to assess how respondents' knowledge and beliefs about HIV varied by geographic (rural, urban) and denominational (Catholic, Lutheran, Pentecostal) factors. Significance levels of p < 0.05, p < 0.01, and p < 0.001 were noted.

Multivariate logistic regression was used to analyze associations between the three outcome variables and demographic characteristics (including denomination), religious beliefs about HIV, and HIV-related knowledge, attitudes, and practices. Two sets of statistical analysis were performed.

First, analysis was done to determine the association between the outcome variables and the demographic factors. For each outcome variable, a nominal logistic regression was performed with covariates being all the demographic factors. The odds ratios (ORs) and p-values of the model were reported for each demographic factor. By construction, these results were adjusted for all other demographic covariates. The regression was performed separately for all three outcome variables.

Next, analysis was done to determine the association of the outcome variables with HIV belief/knowledge factors (religious beliefs about HIV; knowledge, attitudes, and practices relating to HIV and HIV treatment). For each outcome variable and each covariate two nominal logistic regressions were performed: one that was unadjusted, and one that was fully adjusted for all the demographic and HIV belief/knowledge covariates.

All data entry and statistical analyses were performed in JMP 7 (SAS Institute Inc., Cary, NC, 2007). The survey data were entered twice and checked for consistency before analysis.

## Results

### Demographics

The demographic characteristics of the 438 participants in the study are shown in Table 2. Overall, the mean age was 34.5 ± 12.6 years (median of 32 years), 49.1% were male, 58.4% were from an urban area (Arusha), 41.9% had attended at least some secondary school, and 67.3% were married, engaged, or cohabiting. In the sample population 36.2% were Catholic, 37.2% were Lutheran, and 25.7% were Pentecostal.

**Table 2:** Survey demographics (n = 438) grouped by urban or rural setting and survey collection church.

	**TOTAL**	**Urban**	**Rural**	**Catholic Church**	**Lutheran Church**	**Pentecostal Church**
# Respondents	438	256	182	145	167	126

Male (%)	49.1	49.8	48.1	51.4	54.3	39.7

Mean age (stdev) (years)	34.5 (12.6)	33.0 (11.5)	36.6 (13.8)	33.9 (13.8)	37.4 (13.0)	31.4 (9.7)

Some secondary school (%)	41.9	55.6	23.1	47.6	40.5	37.1

Married, engaged, or cohabiting (%)	67.3	62.7	74.0	73.0	73.6	51.3

Attends religious activities more than once a week (%)	62.7	58.8	68.0	54.3	69.3	63.6

Catholic (%)	36.2	55.9	44.1	99.3	6.6	3.2

Lutheran (%)	37.2	52.7	47.3	0.7	91.0	7.9

Pentecostal (%)	25.7	69.0	31.0	0	0.6	88.1

Several respondents identified themselves as belonging to a different denomination than the church they were attending when they completed the survey. This was most noticeable for respondents from the Pentecostal churches: 7.9% were Lutheran and 3.2% were Catholic. In this analysis we consider respondents' to "belong" to the church that they were attending at the time of survey collection.

### Religious Beliefs Related to HIV

Responses to questions concerning religious beliefs related to HIV are shown in the first panel of Table 3. About half of the respondents (53.2%) believed that HIV is a punishment from God; this belief was more prevalent amongst rural respondents (60.8%, p < 0.05). About a third of respondents (34.9%) believed that those who are HIV-infected have not followed the Word of God. This belief was significantly higher amongst rural participants (46.3%, p < 0.001) and differed across denominations (p < 0.01). This belief was lowest amongst Catholic respondents (23.1%). Furthermore, 80.8% of respondents (including 100% of rural Pentecostals) believed that prayer can cure HIV. This belief differed across denominations (p < 0.001) and was lowest amongst Catholic respondents (64.6%) and highest amongst Pentecostal respondents (94.4%). No significant differences were observed between male and female respondents.

**Table 3:** Survey responses.

	Percent Respondents (%)	**TOTAL**	**Urban**	**Rural**	**Cath**.	**Luth**.	**Pent**.	**Male**	**Female**
**1**	Believes HIV is a punishment from God	53.2	47.7 *	60.8 *	47.1	53.2	60.3	49.3	57.2
	
	Believes PLWHA have not followed the Word of God	34.9	26.9 ***	46.3 ***	23.1 **	42.6 **	38.3 **	35.9	34.3
	
	Believes prayer can cure HIV	80.8	81.3	80.0	64.6 ***	83.4 ***	94.4 ***	79.8	82.1

**2**	ARV knowledge	81.4	84.8 *	76.6 *	82.4	82.3	79.3	81.4	81.3
	
	Has been tested for HIV	42.7	51.2 ***	30.3 ***	40.8	40.9	46.8	40.6	44.8
	
	Fears casual contact HIV transmission	56.2	53.6	59.9	56.0	57.6	54.8	54.7	57.4
	
	Of sexually active respondents, % that never uses condoms	55.5	41.6 ***	73.0 ***	42.4 ***	56.4 ***	69.7 ***	45.5 ***	65.8 ***

**3**	Expresses shame-related HIV stigma	54.6	56.0	52.6	51.8 *	49.4 *	64.3 *	52.8	56.4
	
	Willing to disclose to religious community	84.2	83.3	85.4	81.5 *	80.6 *	91.6 *	82.9	85.8
	
	If HIV+, would NOT start ARVs (even) if doctor recommends	6.3	6.8	5.6	2.4 ***	3.3 ***	14.3 ***	5.0	7.6

Responses concerning (1) religious beliefs about HIV, (2) knowledge, attitudes, and practices related to HIV and ARVs, and (3) the outcome variables, grouped by urban or rural setting, survey collection church, and gender. Chi square test significance levels of p < 0.05, p < 0.01, and p < 0.001 denoted by *, **, and ***, respectively.

### HIV and ARV Knowledge, Attitudes, and Practices

Responses to questions concerning HIV and ARV knowledge, attitudes, and practices are shown in the second panel of Table 3. Overall, 81.4% of respondents had some ARV knowledge as measured by the ARV knowledge index. More urban respondents than rural respondents had ARV knowledge (84.8% compared to 76.6%, p < 0.05). Less than half (42.7%) of the respondents had been tested for HIV. Significantly more urban than rural respondents had been tested (51.2% compared to 30.3%, p < 0.001). Of the sexually active respondents, 55.5% replied that they never used condoms. This fraction was smaller in the urban cohort (41.6%, p < 0.001). Catholics were the least likely to say that they never used condoms (42.4%), while Pentecostals were the most likely (69.7%). Females were significantly more likely than males to report that they never used condoms (65.8% vs. 45.5%, p < 0.001). Slightly over half of the respondents (56.2%) expressed fear of casual contact HIV transmission. This percentage did not differ significantly based on site location (urban or rural) or denomination.

### Distribution of Outcome Variables

Responses to the three outcome variables are shown in the third panel of Table 3. Overall, 54.6% of respondents expressed shame-related HIV stigma, 84.2% said that they would disclose their HIV status to their religious community if they became infected, and 6.3% said that they would not begin doctor-recommended ARV treatment if they became HIV-infected. Responses did not differ significantly based on site location (urban or rural) or gender, but did differ based on denomination. A greater percentage of respondents from Pentecostal churches than from other churches expressed shame-related HIV stigma (64.3%, p < 0.05), said they would be willing to disclose their HIV status to the religious community if they became infected (91.6%, p < 0.05), and said that they would not begin ARV treatment if they became HIV-infected (14.3%, p < 0.001)

### Multivariate Analysis

The results of multivariate analyses on the three key indicators in this study (shame-related HIV stigma, intention to disclose to the religious community if HIV-infected, and willingness to begin ARV treatment if HIV-infected) are shown controlling for demographics (Table 4) or a combination of demographics and responses to HIV belief/knowledge questions (Table 5 and Additional file 1).

**Table 4:** Demographic associations with stigma, disclosure, and ARV treatment outcome variables.

	**Expresses Shame-Related HIV Stigma**		**Willing to Disclose**		**Would Start ARVs**	
	*Adjusted OR*	*P-value*	*Adjusted OR*	*P-value*	*Adjusted OR*	*P-value*

Urban	1.18 (0.99–1.62)	0.153	0.88 (0.64–1.21)	0.437	0.83 (0.48–1.39)	0.486

Male	1.05 (0.84–1.31)	0.676	0.98 (0.71–1.33)	0.874	0.96 (0.57–1.63)	0.890

Age <32 years	0.96 (0.78–1.19)	0.734	0.72 (0.53–0.98)	0.036*	0.61 (0.35–1.01)	0.062

Some secondary school	0.81 (0.64–1.01)	0.063	0.92 (0.68–1.24)	0.584	1.8 (1.07–3.29)	0.036*

Married or cohabiting	0.91 (0.72–1.16)	0.472	1.17 (0.83–1.63)	0.336	1.11 (0.64–1.89)	0.714

Attends church >1 per week	1.07 (0.86–1.33)	0.554	1.13 (0.83–1.52)	0.428	0.79 (0.45–1.3)	0.378

Catholic (vs. Pentecostal)	0.86 (0.64–1.15)	0.314	0.85 (0.56–1.31)	0.463	1.9 (0.86–5.18)	0.148

Lutheran (vs. Pentecostal)	0.78 (0.58–1.04)	0.093	0.56 (0.36–0.86)	0.009**	1.28 (0.6–2.88)	0.527

OR adjusted for the eight factors shown in the first column. Significance levels of p < 0.05, p < 0.01, and p < 0.001 denoted by *, **, and ***, respectively. The 95% confidence intervals are shown in parentheses.

**Table 5:** Associations of HIV belief/knowledge factors with stigma, disclosure, and ARV treatment outcome variables.

	**Expresses Shame-Related HIV Stigma**		**Willing to Disclose**		**Would Start ARVs**	
	*Unadj. OR* *(Adj. OR)*	*P-value*	*Unadj. OR* *(Adj. OR)*	*P-value*	*Unadj. OR* *(Adj. OR)*	*P-value*

Believes prayer can cure HIV	1.02 (0.88)	0.899 (0.504)	1.36 (0.99)	0.074 (0.961)	1.06 (2.07)	0.814 (0.171)

Believes HIV is a punishment from God	1.56 (1.46)	<0.001*** (0.008**)	1.17 (1.22)	0.269 (0.306)	0.89 (0.73)	0.570 (0.337)

Believes people with HIV have not followed the Word of God	1.75 (1.92)	<0.001*** (<0.001***)	1.15 (1.15)	0.382 (0.519)	0.85 (1.05)	0.437 (0.876)

ARV knowledge	0.90 (1.01)	0.519 (0.949)	0.96 (1.11)	1.00 (0.677)	1.98 (2.98)	0.003** (0.002**)

Has had HIV test	1.00 (1.00)	1.00 (0.989)	1.41 (1.66)	0.019* (0.020*)	0.97 (1.04)	0.887 (0.913)

Fears casual-contact HIV transmission	1.29 (1.11)	0.011* (0.464)	0.87 (0.90)	0.320 (0.600)	1.28 (1.52)	0.229 (0.173)

Never uses condoms	1.00 (0.83)	1.00 (0.233)	1.35 (1.38)	0.060 (0.116)	0.56 (0.70)	0.027* (0.329)

The first OR shown is unadjusted. The second OR shown (in parentheses) is adjusted for the HIV belief/knowledge factors listed in the first column as well as for the demographic factors shown in Table 4. Significance levels of p < 0.05, p < 0.01, and p < 0.001 denoted by *, **, and ***, respectively. The 95% confidence intervals are reported in Additional file 1.

#### 1. Factors Associated with Shame-Related HIV Stigma

Shame about HIV was strongly associated with certain religious beliefs. After adjusting for demographic and HIV belief/knowledge factors, respondents who believed that HIV is a punishment from God or that people with HIV have not followed the Word of God were significantly more likely to express shame-related HIV stigma than those who did not (OR 1.46, p < 0.01 and OR 1.92, p < 0.001).

#### 2. Factors Associated with Intention to Disclose to the Religious Community if HIV-Infected

Respondents who were less than 32 years old were less likely to say that they would disclose their HIV status than older respondents (OR 0.72, p < 0.05). Intention of disclosure to the religious community was higher amongst people who had been tested for HIV (OR 1.66, p < 0.05). Non-denominational religious beliefs about HIV were not significantly associated with people's intentions to disclose to the religious community.

#### 3. Factors Associated with Willingness to Begin ARV Treatment if HIV-Infected

Demographic factors and ARV knowledge factors were significantly associated with respondents' receptiveness to ARV treatment. Religious beliefs relating to HIV were not significantly associated. Having completed at least some secondary schooling significantly increased the likelihood that a respondent would be willing to begin treatment (OR 1.80, p < 0.05). Having some degree of ARV knowledge as measured by the ARV knowledge index described previously also significantly increased the likelihood that a respondent would be willing to begin treatment (OR 2.98, p < 0.01). The belief that prayer can cure HIV was not significantly associated with respondents' hypothetical willingness to begin ARV treatment if they became HIV-infected.

#### 4. Associations Between Outcome Variables and Denomination

When adjusted for demographic factors, Lutherans were significantly less likely than Pentecostals to say that they would disclose to their religious community if they became HIV-infected (OR 0.56, p < 0.01), and tended to be less likely to express shame-related HIV stigma (OR 0.78, p < 0.1).

While the p-values were not significant in the comparison of the willingness of congregants from different denominations to start ARVs if they became HIV-infected, the data suggest that there may be denominational differences. Of the 25 people who replied that they would not start ARVs if found HIV-infected, 17 were Pentecostal (for reference, Pentecostals made up only 25.7% of the total sample population). Thus, even though the p-values were insignificant – probably due to the fact that there were so few "No" responses to the question about starting ARVs – the overrepresentation of Pentecostals in the group that would decline treatment is still an interesting observation, especially in the context of other belief results.

## Discussion

This study investigated how the religious beliefs of churchgoers in Tanzania relate to attitudes about HIV and HIV treatment. The survey data suggest that while religion plays a significant role in people's attitudes about HIV and PLWHA, non-religious factors such as education level and basic knowledge about ARVs are the main predictors of whether people would begin ARV treatment if they were found to be HIV-infected.

### Shame-Related HIV Stigma

We examined the shame dimension of HIV stigma as a key outcome variable in order to provide insight into how specific religious beliefs are associated with feelings of HIV-related shame. Our findings suggest that religious beliefs play a large role in shaping people's perspectives about HIV and PLWHA. Shame about HIV was significantly more prevalent amongst people who attach religiously-based blame to PLWHA. In the multivariate analysis people who believed that HIV is a punishment from God or that PLWHA have not followed the Word of God were significantly more likely than those who did not to express shame-related HIV stigma. This illustrates how religious beliefs can strongly influence the shame-related HIV stigma experienced by PLWHA. It also suggests that religious leaders may have the power to address shame-related HIV stigma in their congregation, thereby enhancing the faith community's potential to assist in coping with HIV rather than contributing to the social stress and mental health co-morbidities.

Religious beliefs and organizations have the potential to either mitigate or exacerbate shame-related HIV stigma. Disclosure of HIV-status to pastors or other members of the religious community can facilitate emotional healing and support, and religion can provide mechanisms for coping and hoping [22,21]. However, the present finding that religious beliefs about HIV are strongly associated with shame stigma illustrates that religion can also be a significant source of negative perceptions about HIV and PLWHA. Beliefs such as these have likely contributed to the discrimination that PLWHA have reported experiencing in church settings [14]. The connection between religion, HIV-related shame and behavior has also been described in a study of HIV-infected patients in the United States. In a cohort of hospitalized HIV-infected patients, those who believed that HIV was a punishment from God or who felt guilty about having HIV were more likely to fear death and to not have discussed resuscitation status with their physician [25]. Together, these findings illustrate how religion mediates HIV-related shame in several ways, and suggest that the shame dimension of stigma may be reduced by appropriate interventions in religious communities.

### Disclosure to the Religious Community

Previous research has documented many personal and community-level factors that influence people's decisions to disclose their HIV status to family members and sexual partners [18,19]. In the present study, two measures of disclosure intentions (to pastor and to congregation) were combined to form a religious community disclosure index. This index was used to assess what kinds of religious or sociodemographic factors and environments are conducive to disclosure intentions in religious settings. Overall, a high percentage of respondents (84.2%) said that they would disclose to the religious community if they became HIV-infected.

After adjusting for demographic and HIV belief/knowledge factors, the odds of disclosure willingness were almost three times higher for people who had been tested for HIV than for people who hadn't. This may suggest that people who are willing to confront the possibility of being HIV-infected are also willing to make their status known to the religious community, independently of other religious beliefs or sociodemographic characteristics. Education level, urban/rural residence status, and ARV knowledge were not significantly associated with willingness to disclose. This implies that the act of getting tested facilitates disclosure intentions, rather than the educational and access factors that are associated with testing. It is also possible that respondents who had been tested and were HIV-negative answered the disclosure intention questions overly positively due to the knowledge that they were HIV-negative and would not be faced with disclosure situations in the immediate future.

It is interesting to note that Pentecostals were significantly more likely than Lutherans to say that they would disclose their status to their religious community if they became HIV infected, even though they also tended to be more likely to express shame-related HIV stigma. One explanation for this somewhat counterintuitive trend in disclosure intentions may be that Pentecostals have a more emotive worship style, with the expectation of disclosure of sins and a pietistic understanding of sin and forgiveness.

Data about disclosure intention trends within religious environments are relevant to HIV treatment programs and strategies. Although disclosure of HIV status can be difficult due to fears about stigmatization, abandonment, and blame [20], disclosure can improve disease prognosis by facilitating initiation of and adherence to ARVs [28,29]. In a study of Tanzanians enrolled in an ARV treatment program, subjects who disclosed their HIV status to a person other than a health care provider were more likely to adhere to their medications and less likely to progress to virologic failure than subjects who did not [28]. This suggests that disclosure to pastors or other members of the religious community can be important in assisting in ARV adherence, and reinforces the need to address the association between shame-related HIV stigma and religious beliefs about HIV.

Church members' attitudes about disclosing their HIV status to the religious community are also quite relevant to church policy. Some churches require couples to present their HIV testing results to their pastor before they can be married. Mandatory (or in some cases heavily encouraged) HIV testing and disclosure of results to religious leaders before marriage has been praised as a mechanism to slow the spread of HIV, but has also been accused of being discriminatory and imposing [12,13]. Further exploration of the intersection between religion and disclosure of HIV status will help clinicians as well as church leaders to form effective treatment programs and policies.

### Willingness to Begin ARV Treatment if HIV-Infected

There is reason to believe that uptake, adherence and continuation of ARVs may be influenced by religious beliefs. In a study in Uganda, a small but important number of patients (6 out of 558) discontinued their ARV treatment because they believed that they had been cured of HIV through prayer [27]. In the present study we found that, when adjusted for confounding factors, belief in the healing power of prayer was not significantly associated with a person's hypothetical willingness to begin ARV treatment if they became HIV-infected. Instead, willingness to begin ARVs was associated with age, education, and ARV knowledge. The belief that prayer could cure HIV (80.8% of respondents) was not mutually exclusive with respondents' willingness to begin ARV treatment if HIV-infected (93.7% of respondents). This suggests that while a small fraction of people may decline ARV treatment because of their belief in the healing power of prayer, in the majority of cases religious beliefs about HIV healing pose no obstacle to the acceptance of medical treatment.

Coexistence of the belief that prayer can cure HIV and the willingness to begin ARV treatment if HIV-infected may be partially explained by considering the different conceptualizations that people have of the AIDS virus. A qualitative interview study found that members of a Neo-Pentecostal church in Dar Es Salaam, Tanzania believed that the virus causing AIDS could either be a regular medical virus, or a spirit disguised as a virus [11]. They believed that if an HIV-infected person was afflicted by the spirit disguised as a virus, then it could be cast out by pastors and prayers, and the person would be cured of HIV. However, if the virus was a normal medical virus, then the spiritual healing process would not necessarily be successful in eradicating the virus. Thus in some cases prayer is seen as sufficient for healing, while in others medicine is needed.

Similarly, it is plausible that respondents had a variety of conceptualizations of how prayer could cure HIV. For example, some may view the availability of ARVs as God's answer to a prayer for a cure (or an acceptable treatment option), and therefore consider prayer and ARVs to be closely related methods of treating HIV. It is also likely that some respondents would try multiple treatment methods – prayer, ARVs, traditional medicine, etc. – in hopes of treating their disease using any means possible. From this pragmatic viewpoint it is easy to see how the belief in prayer healing, willingness to begin ARVs, and any number of other treatment beliefs can coexist.

The recent growth of Pentecostal churches has been partially attributed to the attraction of people, especially those from low socioeconomic brackets, to the possibility of prayer healing in Pentecostal churches and testimonials from members who have allegedly been cured of HIV and other illnesses by prayer [8,11]. In the present study a greater fraction of Pentecostal church members than Catholic or Lutheran church members believed that prayer can cure HIV and said that they would refuse ARV treatment. However, the lack of association in the multivariate regression between belief in prayer healing and willingness to start ARVs implies that most people would not reject ARVs in order to rely solely on prayer healing. Instead, the strong correlation between lack of ARV knowledge and hypothetical refusal of ARV treatment suggests that respondents who refused ARVs did so because they lacked information about how to access ARVs and what medical benefits taking ARVs could bring. This lack of information could result in the perception that ARVs are not a tenable treatment option.

Although only a small fraction of the sample (6.3%) said that they would decline ARV treatment if they became HIV-infected and doctors recommended starting it, it is informative to look closely at the characteristics of this group in order to begin to understand what factors motivate their (hypothetical) decision to decline treatment. Respondents who would decline treatment were more likely to lack basic knowledge about ARV access and medical benefits of ARVs and not have attended any secondary schooling. These results reinforce the need for HIV outreach, testing, counseling, and treatment programs focusing on people with low education level or limited exposure to information about ARVs.

Since ARV knowledge is a predictor of willingness to begin ARV treatment, it is interesting to examine the relationship between ARV knowledge and religion. Although no significant difference in ARV knowledge was observed between denominations in the present study, several previous multivariate regression analyses have suggested that religious affiliation is indeed correlated with HIV/AIDS knowledge. A study of Ghanaian women found that respondents who identified themselves as Christians were more knowledgeable about modes of HIV transmission than women who followed African traditional religions or were not religiously affiliated [8], and a study of church members in Mozambique found that members of "mainline" (Roman Catholic, Presbyterian) churches had more exposure to HIV/AIDS information through church activities and more basic knowledge about HIV/AIDS than members of "healing" (Pentecostal) churches [9]. These observations reinforce the importance of considering the relation between HIV or ARV knowledge, religious environment, and attitude towards HIV treatment when forming HIV treatment and outreach programs.

### Limitations

There were several general limitations in this study. First, only one congregation was sampled for each denomination in each region. Due to this convenience sampling it is likely that some variations within the population were overlooked. It is also important to note that only the three main Christian denominations in Tanzania were included in this exploratory study. Although many of the survey questions addressed general aspects of spirituality and religion that were not specific to Christianity, future studies about HIV beliefs in the Muslim and traditional religion communities would be useful.

Second, the congregants who participated in the survey were also convenience sampled and information about non-respondents was not collected. At the Pentecostal church in Arusha surveying was done during the Tuesday evening fellowship meeting instead of during the standard sampling time at Sunday morning services. If the population attending the fellowship meeting different from the general congregation then the responses collected from this group may have been biased.

Third, the surveys were collected in the presence of pastors after church services. This may have introduced bias in the responses. We tried to minimize this bias by having local assistants explain the project and distribute and collect the anonymous surveys.

Fourth, the survey questions that were used to assess shame, disclosure, and ARV treatment were by necessity phrased in the hypothetical sense (e.g. "*If *you were HIV-infected, would you..."). Although the responses to these questions are useful for understanding how people claim they would react to certain situations, the results cannot be treated as actual enacted behaviors. On the other hand, it is possible that some of the survey respondents were themselves HIV-infected. The survey did not ask respondents for their HIV status because the benefits of collecting this information did not outweigh the potential harm that could be done if a breach in privacy occurred during the survey administration. However, it is important to note that the views of HIV-infected respondents on some issues are most likely different from the views of the uninfected population. If HIV-infected respondents were over represented in any of the sample groups in this study the results may be skewed.

Finally, not all of the questions on the survey were previously validated.

## Conclusion

Religious beliefs strongly influence the way many Tanzanians think about HIV/AIDS. A significant percentage of those surveyed believed that people who are HIV-infected have not followed the Word of God, that HIV is a punishment from God, and that through prayers it can be cured. Shame-related HIV stigma was strongly correlated with religious beliefs about punishment from God and following the Word of God. This held even after adjusting for demographics and HIV belief/knowledge factors. Understanding which religious beliefs are associated with shame-related HIV stigma and assessing their prevalence in different congregations and demographic groups can focus outreach campaigns and theological discussions to reduce HIV stigma among religious audiences. Respondents' intentions of disclosing their status to the religious community (pastor, congregation) if they became HIV-infected was primarily associated with non-religious factors in the multivariate analysis. Gaining an understanding of how church members look to their religious community for guidance and support in matters related to HIV can help to direct collaborative efforts between church leaders and clinicians or HIV educators.

Although religious beliefs were significantly associated with shame-related HIV stigma, their impact on people's willingness to receive medical treatment for HIV was minimal. The vast majority of respondents believed that prayer could cure HIV, but said they would still accept ARV treatment if they became HIV-infected. When demographics and HIV belief/knowledge factors were controlled for in the multivariate analysis there was no significant association between a person's belief that prayer can cure HIV and their willingness to accept ARV treatment. The hypothetical decision to refuse ARV treatment was instead strongly associated with factors such as lower education level and lack of basic ARV knowledge.

Together, these findings suggest that religious beliefs should be incorporated and addressed by interventions to reduce HIV stigma related to shame, while policies designed to improve ARV treatment and adherence should focus primarily on addressing sociodemographic factors. Enhanced educational initiatives focused within faith communities may help to reduce stigma and enhance disclosure, contributing to improved social supports for coping with HIV and the potential to increase ARV adherence.

## Abbreviations

AIDS: Acquired immune deficiency syndrome; ARV: Antiretroviral; HIV: Human immunodeficiency virus; PLWHA: People living with HIV/AIDS.

## Competing interests

The authors declare that they have no competing interests.

## Authors' contributions

JZ conceived of the study, supervised the data collection, and performed the statistical analysis. YY participated in the study design, data collection and drafted the manuscript. MJ participated in survey design and data collection. MW participated in the study design. JO participated in statistical analysis. NT participated in the study design. All authors read and approved the final manuscript.

## Pre-publication history

The pre-publication history for this paper can be accessed here:



## Supplementary Material

Additional file 1 **Supplementary Table 1.** Associations of HIV belief/knowledge factors with stigma, disclosure, and ARV treatment outcome variables, with 95% confidence intervals. Odds ratios adjusted for the HIV belief/knowledge factors listed in the first column as well as for the demographic factors shown in Table 4, with 95% confidence intervals shown in parentheses. Significance levels of p < 0.05, p < 0.01, and p < 0.001 denoted by *, **, and ***, respectively. Click here for file
