# Supplementary material for: Religion and HIV in Tanzania: influence of religious beliefs on HIV stigma, disclosure, and treatment attitudes
Source: BMC Public Health. 2009 Mar 4;9:75. doi: 10.1186/1471-2458-9-75 (PMC2656538; doi:10.1186/1471-2458-9-75)
Supplement: Additional file 1 — Supplementary Table 1. Associations of HIV belief/knowledge factors with stigma, disclosure, and ARV treatment outcome variables, with 95% confidence intervals. Odds ratios adjusted for the HIV belief/knowledge factors listed in the first column as well as for the demographic factors shown in Table 4, with 95% confidence intervals shown in parentheses. Significance levels of p < 0.05, p < 0.01, and p < 0.001 denoted by *, **, and ***, respectively. [file 1471-2458-9-75-S1.doc]

**Supplementary Table 1. Associations of HIV belief/knowledge factors with stigma, disclosure, and ARV treatment outcome variables, with 95% confidence intervals.**

|  | **Expresses Shame-Related HIV Stigma** | | **Willing to Disclose** | | **Would Start ARVs** | |
| --- | --- | --- | --- | --- | --- | --- |
|  | *Adjusted OR*  *(95% CI)* | *P-value* | *Adjusted OR*  *(95% CI)* | *P-value* | *Adjusted OR*  *(95% CI)* | *P-value* |
| Believes prayer can cure HIV | 0.88  (0.6-1.28) | 0.504 | 0.99  (0.61-1.57) | 0.961 | 2.07  (0.67-5.81) | 0.171 |
| Believes HIV is a punishment from God | 1.46  (1.11-1.95) | 0.008** | 1.22  (0.83-1.81) | 0.306 | 0.73  (0.37-1.35) | 0.337 |
| Believes people with HIV have not followed the Word of God | 1.92  (1.41-2.66) | <0.001*** | 1.15  (0.76-1.77) | 0.519 | 1.05  (0.57-2.01) | 0.876 |
| ARV knowledge | 1.01  (0.97-1.44) | 0.949 | 1.11  (0.69-1.71) | 0.677 | 2.98  (1.53-6.18) | 0.002** |
| Has had HIV test | 1.00  (0.75-1.34) | 0.989 | 1.66  (1.10-2.59) | 0.020* | 1.04  (0.55-2.01) | 0.913 |
| Fears casual-contact HIV transmission | 1.11  (0.84-1.48) | 0.464 | 0.90  (0.61-1.32) | 0.600 | 1.52  (0.84-2.87) | 0.173 |
| Never uses condoms | 0.83  (0.61-1.13) | 0.233 | 1.38  (0.92-2.09) | 0.116 | 0.70  (0.22-1.38) | 0.329 |

Odds ratios adjusted for the HIV belief/knowledge factors listed in the first column as well as for the demographic factors shown in Table 4, with 95% confidence intervals shown in parentheses. Significance levels of p<0.05, p<0.01, and p<0.001 denoted by *, **, and ***, respectively.
